# Field Epidemiology: Fit for the future

**DOI:** 10.2807/1560-7917.ES.2023.28.36.2300347

**Published:** 2023-09-07

**Authors:** Susan Hahné, Charlotte Hammer, Alma Tostmann, Jane Whelan, Christopher Williams

**Affiliations:** 1 National Institute for Public Health and The Environment (RIVM), Bilthoven, The Netherlands; 2 University of Cambridge, Cambridge, United Kingdom; 3 EPIET Alumni Network (EAN) Board; 4 Radboud university medical centre, Nijmegen, The Netherlands; 5 EpiSmart, Amsterdam, The Netherlands; 6 Public Health Wales, Cardiff, United Kingdom; * These authors contributed equally

**Keywords:** Epidemiologists, Epidemiology, Field epidemiology, Disease Outbreaks, Surveillance, Public Health, Workforce, Continuous professional development

## Abstract

In recent years, field epidemiologists have embraced rapidly evolving digital tools, data sources and technologies, and collaborated with an ever-growing field of scientific specialisms. The COVID-19 pandemic put field epidemiology under unprecedented demand and scrutiny. As the COVID-19 emergency recedes, it is timely to reflect on the core values of our profession and the unique challenges and opportunities that lie ahead. In November 2022, alumni of the European Programme for Intervention Epidemiology Training (EPIET) and the European Public Health Microbiology (EUPHEM) training programme celebrated 25 years of EPIET, and the present and future of field epidemiology was discussed. The output was recorded and qualitatively analysed. This Perspective reflects the authors’ interpretation of the discussion. We should reaffirm our commitment to field epidemiology’s core strengths: competence and rigour in epidemiology, surveillance, outbreak investigation and applied research, leading to timely and actionable evidence for public health. Our future success will be defined by an ability to adapt, collaborate, harness innovation, communicate and, ultimately, by our tangible impact on protecting and improving health.

## Background

The COVID-19 pandemic highlighted the urgent need for more epidemiologists in the field, better training and increased investment [1]. A greater emphasis on leadership, communication, interpersonal skills and specialist training in emergency response has also been called for during training [2]. As global public health emergencies become more common and complex, we believe that the role of the field epidemiologist needs to be more clearly defined [3], but also to evolve. We therefore reflect on the evolution of the profession of field epidemiology and share our views on emerging challenges and opportunities and our vision for the future.

## Methods

Our views are informed by a qualitative analysis of output from a workshop held in November 2022 in Stockholm, Sweden, to celebrate the 25-year anniversary of the European Programme for intervention Epidemiology Training (EPIET). Workshop participants were alumni and fellows of EPIET and of the European Public Health Microbiology (EUPHEM) training programme. We collected responses to three broad guiding questions on the future of field epidemiology in short note format using sticky notes during this workshop. Data were analysed using grounded theory approaches, allowing the themes to emerge inductively. Please see the Supplement for details on the qualitative method used.

## The Past: The traditional role of field epidemiologists

‘Field’, ‘intervention’ or ‘applied’ epidemiology all imply epidemiological investigations initiated in response to urgent public health problems, typically caused by infectious diseases. The investigative team does much of its work in the field (i.e. outside the office or laboratory) where real-world events are observed through ‘field work’ such as site visits, interviewing people with suspected symptoms and gathering surveillance data from healthcare providers [4,5]. Historically, ‘field work’ was conducted by medical doctors in the community, however, the professional background of field epidemiologists is now diverse, including health and social scientists, nurses, veterinarians and environmental health experts. Field epidemiology is an interdisciplinary practice and liaising with stakeholders including policymakers, public health practitioners and other scientists is crucial at all stages, from defining the study aims to appropriately interpreting and communicating results. Another key role of field epidemiologists is to pragmatically synthesise diverse pieces of data and information into actionable evidence and to communicate tailored public health messages effectively to stakeholders and the general population [6,7]. This holistic approach to evidence generation and dissemination is necessary to allow for timely, high-quality public health decision-making.

The direct application of results for public health action differentiates field epidemiology from academic epidemiology, which is generally more focussed on furthering scientific knowledge and discourse, and where studies may take years. To deliver timely, actionable evidence, speed must sometimes be prioritised over rigour. This means that a field epidemiologist needs robust methodological knowledge in outbreak investigation, surveillance, operational research and communication [8], and the skills to appropriately interpret imperfect data to show empirically that the situation is resolved or improving. Training for this highly specific skillset has been replicated in successful field epidemiology training programmes around the world, with graduates represented in local, regional, national and international public health bodies globally [9].

## Evolution of the profession of field epidemiology

Over the years, field epidemiology has evolved, and the skillset of field epidemiologists has been adapted accordingly. Originally, investigations used in-person, paper-based interviews to evaluate risk and exposures [10-12]. Data collection and linkage has since been modernised through electronic survey tools, customer data and food tracing [10], facilitating remote, office-based investigations. Large datasets of electronic health records are increasingly used for data collection purposes in infectious disease surveillance. A promising recent addition to the toolbox for field epidemiology and microbiology is the extended use of wastewater for surveillance and early detection of pathogens [13]. These tools and new sources of data have, to a certain extent, helped us overcome a decline in the response rates for questionnaire surveys and surveillance forms [14].

Another key development is the rapid evolution and application of genomics and bioinformatics, powerful tools especially when combined with epidemiological information. This has enabled the identification and investigation of outbreaks that may otherwise remain concealed, especially when cases are diverse or dispersed, requiring remote investigation across a large area. It also facilitates attribution of infections to their source [15,16]. During the Ebola outbreak response in 2014 and 2015 in West Africa, genomics assisted in our understanding of transmission, although intensive field work was still essential in identifying transmission patterns and tailoring control measures [17]. Notwithstanding these major technological advances, basic descriptive field epidemiology, as applied for nearly 200 years, remains one the most powerful tools for actionable insights [18,19].

## The Present: Recent challenges

The emergence of severe acute respiratory syndrome coronavirus 2 (SARS-CoV-2) and its rapid global spread put field epidemiology under unprecedented demand and scrutiny. As public and political concern grew, the need for data and information escalated. Mass testing, driven by the World Health Organization (WHO)’s call to “*Test, test, test*” [20], meant that surveillance departments in public health institutions had to gain rapid access to (often sparse) data on testing and harness it systematically to produce reliable indicators of disease spread. Despite insufficient data, field epidemiological studies provided answers to urgent questions (e.g. transmission routes) to inform recommendations. Data science and modelling were applied to problems such as the assessment of variant growth, vaccine effectiveness and prediction of the resurgence of pre-pandemic respiratory infections [21,22]. Mathematical modelling was essential to guide and justify decisions on major societal public health interventions such as enforced physical distancing and to predict health service needs. All of this work was vital, particularly in the early stages of the pandemic when surveillance data were incomplete and key infection parameters were unknown [23,24]. Research output in the fields of virology, vaccinology, epidemiology, diagnostics, immunology, behavioural science, data science, mathematical modelling and clinical medicine grew exponentially as the wider scientific community mobilised and collaborated in a way that was unparalleled before the pandemic [25]. The rapid evolution of knowledge and the resulting complexity, sometimes with divergent or conflicting findings, may have contributed to public uncertainty and political debate. With so many different voices and perspectives, conveying a unified public health message was challenging. Field epidemiologists working in public health agencies and/or as members of government advisory bodies were usually not in a position to voice their personal opinion, even though they were well informed about what was needed to protect public health. Field epidemiology investigations were conducted in specific situations [26-28] but the rapid and multimodal spread of SARS-CoV-2, along with underlying risks such as inequality, poverty, ethnicity and age structure, meant that studying the main transmission settings and contexts was challenging and often not a priority. The increased day-to-day workload also meant that immediate publication of results in journals and as pre-prints was deprioritised. For these reasons, public opinion was often influenced by experts outside of public health agencies, whereby field epidemiology was relatively less visible.

While the COVID-19 pandemic has been a very particular public health emergency, we continue to face a multitude of challenges globally, including the consequences of climate change, geopolitical instability, war and migration [29]. Field epidemiology is essential for effective emergency response and has the potential to contribute to mitigating instability by improving public health and reducing socioeconomic inequalities. It is therefore timely to reflect on the core values of the profession and the unique challenges and opportunities that lie ahead for field epidemiology if we are to maximise our impact in the prevention and control of infectious diseases.

## Defining our future success

We argue that a new model to future-proof field epidemiology is required, in which the unique strengths of the profession are sustained and developed and are complemented by our openness and willingness to embrace new challenges and collaborations. This will require a dialogue within our profession; here we propose some ideas, summarised in the Figure.

**Figure:**
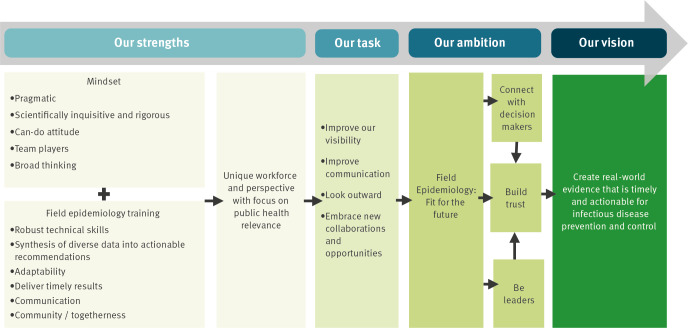
Framework summarising field epidemiologists’ strengths, ambitions and vision

To sustain our strengths, we should be advocates for competence and rigour in field epidemiology practice. Robust study design, data collection, analyses, interpretation and communication should remain at the core of field epidemiology training and continuous professional development. Regardless of professional background, the shared experience of field epidemiology training fosters collective competence, mutual trust and builds a strong, durable and diverse international network.

Maintaining a field perspective also remains important, whereby all necessary sources of information are used and distilled into answers that address public health priority questions. We should value these skills and our collective experience highly and not focus solely on data-driven approaches, instead using these as complementary or integrated methods.

At the same time, we need to adopt an outward-looking perspective and embrace new methods, collaborations and ways of working. This requires us to cultivate and expand our network to include a wide range of professionals. Previously, we worked mostly with public health experts, clinicians, mathematical modellers, statisticians and behavioural experts. We now must expand our vocabulary, be comfortable with the terminology of a growing and diverse range of other disciplines, and leverage the expertise of data scientists, geneticists, bioinformaticians, infodemiologists, privacy experts, lawyers, artificial intelligence experts, IT-experts and software developers. How best to achieve this depends on the local situation and resources available, but some reorganisation of epidemiology departments in public health institutes is probably required. More focus on continuous professional education on leadership and management was one of the recommendations from the recent member survey of the EPIET Alumni Network, as during the COVID-19 pandemic, many field epidemiology and public health microbiologists felt that these were among much-needed yet underdeveloped professional skills [30].

## The strength of establishing networks

Good collaboration starts with a clear understanding of the roles and responsibilities among an expanding team of professionals involved, to prevent gaps and duplication of effort. For mathematical modellers, field epidemiologists provide essential, real-world data and perform studies to estimate key epidemiological parameters to input in their models. For data scientists, our knowledge of the origin of data and its strengths, limitations and related biases ensures that conclusions are valid and grounded in reality. For behavioural scientists, we can inform the definition of the relevant research questions. An important and related issue, however, is to identify who bears ultimate responsibility for the quality of the end-product: *Where does the buck stop?*

Firstly, it is essential that we ourselves have a good understanding of what the profession of field epidemiology can offer, and that we advocate this. While working at the interface between many specialties involved in addressing public health problems, we need to cherish our distinct macro-perspective and skills, to effectively synthesise wide-ranging pieces of evidence, as well as a pragmatic and flexible ‘can-do’ mindset and use of practicable methods inherent in field epidemiology. These characteristics are essential to deliver timely and actionable recommendations. Field epidemiologists at all levels can further grow the reputation of the profession, promoting its findings and recommendations through active engagement, advocacy and training others about our role.

## Future outlook

The profession of field epidemiology will benefit from looking outward and proactively engaging with the wider scientific community, policymakers and the general public to articulate and assert our public health message and advocate for our profession. In turn, this will equip us to connect more effectively with decision makers, embrace positions of leadership, and build public and professional trust and influence. The future success of the profession of field epidemiology will be defined by the quality of our core skills and competencies, but also by our ability to adapt, collaborate and harness innovation. This balance is worthy of renewed dialogue within our profession if we are to continue to have tangible impact on protecting and improving health.

## Note

This Perspective represents the authors’ analyses and interpretation of discussions that took place with fellows and alumni of the European Programme for Intervention Epidemiology Training (EPIET) and the European Public Health Microbiology (EUPHEM) training programme on 26 November 2022 in Stockholm, Sweden, at the 25-year celebration of the EPIET programme. This perspective reflects the views of the authors and not necessarily those of their employers.

## Supplementary Data

214000 Supplement
