# Supplementary material for: Field Epidemiology: Fit for the future
Source: Euro Surveill. 2023 Sep 7;28(36):2300347. doi: 10.2807/1560-7917.ES.2023.28.36.2300347 (PMC10486192; doi:10.2807/1560-7917.ES.2023.28.36.2300347)
Supplement: Supplement [file 23-00347_HAHNE_Supplement.pdf]

Supplemental information: Qualitative methods used.

This supplementary material is hosted by *Eurosurveillance* as supporting information alongside the article 'Field Epidemiology: fit for the future', on behalf of the authors, who remain responsible for the accuracy and appropriateness of the content. The same standards for ethics, copyright, attributions and permissions as for the article apply. Supplements are not edited by *Eurosurveillance* and the journal is not responsible for the maintenance of any links or email addresses provided therein.

Members of the EPIET Alumni Network (EAN) and current EPIET and EUPHEM fellows were invited to participate in a 1.5 hour working session hosted at the Public Health Agency, Sweden. Attendance was possible either in person or online. Three opening questions with guiding text were proposed by volunteer members of EAN, then reviewed and agreed with EAN board members:

1. What sets EPIET and EUPHEM alumni apart from other public health professionals involved in outbreak investigation, surveillance and research of infectious diseases?
2. What aspects do we need to evolve to ensure that interventional epidemiology is at the centre of decision making in the future?
3. What does success look like for our profession?

Participants were invited to discuss each question and respond in real time either verbally or in writing. The session was digitally recorded and sticky notes were used to capture emerging insights in writing. The qualitative output was analysed using a grounded theory approach, allowing the themes to emerge inductively, directly from the data itself. The raw data were manually coded and through an iterative process, concepts and categories were developed to arrive at an overarching theory. Qualitative findings were supplemented with descriptive quantitative data. The outcome was presented in the form of a figure, with added narrative for more detailed understanding.

Data analysis

Our aim was to conduct a comprehensive analysis in line with qualitative methodology which seeks to go beyond a mere narrative summary. Hence, we used a Grounded Theory approach for the analysis of the notes from the participants. Grounded Theory was particularly appropriate for this assessment as it can be seen as one of the most empirically-based qualitative methodologies (Glaser & Strauss 1967). Grounded Theory differs from other methodologies, insofar as no existing theoretical framework is used to guide the analysis, but rather the results emerge from the data itself leading to the development of theory grounded in the data (Allan 2003). We coded the notes, and through an iterative process developed concepts and categories to arrive at an overarching framework and call to action. The development of concepts was initially done individually for the three questions we asked participants and we finally sought to arrive at one framework, taking into account concepts and categories from all data sources by way of triangulation and integration.

References

Allan G. A Critique of Using Grounded Theory as a Research Method. *Electronic Journal of Business Research Methods*. 2003. 2(1):1-10.

Glaser B, Strauss A. The Discovery of Grounded Theory: Stretegies for Qualitative Research. Chicago: Aldine; 1967.
